# Dentists in the Army

**Published:** 1898-07

**Authors:** 


### EDITORIAL.

### Dentists in the Army.

This subject has been somewhat discussed in the journals and meetings of dentists for a number of years past, and, in the main, little has been accomplished, except drawing the attention of the public to the subject.

The education of the public in regard to the functions and possibilities of dentistry is being rapidly and widely disseminated.

Dentists are now called to occupy positions in hospitals, in asylums, in schools and in other positions that a little while ago was scarely anywhere thought of. Perhaps calls to such positions was in the first place regarded as only experimental. In the last few years it has come to be esteemed of such importance that the services of the dentist in many such places is held as not only important, but essential to those receiving their services. And it is a fact, in many instances, that the authorities in many of these places would not think of being without their services. Dentists are oftentimes called in consultation in hospitals and in most institutions where medical treatment is demanded.

Nowhere, perhaps, in public departments, would the services of the dentist be more important than in the army and navy of our country. They would certainly be the means of mitigating great suffering in many instances. Their services would result in saving and rendering useful thousands and perhaps hundreds of thousands of teeth that have thus far been ruthlessly destroyed, and of rendering their possessors, our soldiers and seamen, better able to masticate the heavy, rough food upon which they generally subsist, and thus maintaining them in a better condition for performing their work as warriors.

In enlisting the men for either the army or navy the teeth are very carefully examined by the enlisting surgeon, and any especial deficiency found, even the loss of a tooth from the front part of the mouth, is sufficient for rejection of the applicant. This scrutiny and care seems a little inconsistent with the utter neglect of the Government to provide any preventive or remedy for diseases of the teeth and mouth, that are so likely to occur in both army and navy life.

Dentists are capable of, and do render to those in civil life, invaluable services, even to warding off or checking diseases, and maintaining better health, and prolonging life.

Are not those who risk their lives, health, and all the comforts of civilized life for the protection of lives, property and the country, entitled to every comfort that can be had for their health, strength and comfort.

There ought to be at least one thoroughly competent dentist in each regiment, who, in addition to his special work, might be of great value to the army surgeon and physician. The attainments of a thoroughly capable dentist are such as to make him of much importance in consultation, and suggestively in regard to many diseases and also in regard to hygiene and all that pertains to it.

If State or national sanction or legislation is necessary for the introduction of dentists in the army and navy, it should be had at once. If, however, there is any authority in the hands of the President of the United States or any other functionary, it should be exercised at once, and an opportunity afforded for capable dentists to enter the army with suitable rank, that they might render the services so greatly needed in multitudes of cases.

These suggestions are given upon some familiarity with and observation upon the teeth and mouths of those who have been in the army for a number of years.

The rule is that the teeth of such persons, through neglect and abuse, are very defective and deficient in the work they are designed to accomplish. Shall not the patriots who go forth to defend the people of our country have all the care and attention that a loving people can bestow upon them?

